# Examining Rural Food-Insecure Families’ Perceptions of the Supplemental Nutrition Assistance Program: A Qualitative Study

**DOI:** 10.3390/ijerph17176390

**Published:** 2020-09-02

**Authors:** Lindsey Haynes-Maslow, Annie Hardison-Moody, Megan Patton-Lopez, T. Elaine Prewitt, Carmen Byker Shanks, Lauri Andress, Isabel Osborne, Stephanie Jilcott Pitts

**Affiliations:** 1 Department of Agricultural & Human Sciences, North Carolina State University, Raleigh, NC 27659, USA; annie_hardison-moody@ncsu.edu; 2 Division of Health & Exercise Science, Western Oregon University, Monmouth, OR 97361, USA; pattonlm@wou.edu; 3 Department of Health Policy and Management, University of Arkansas for Medical Sciences, Little Rock, AR 72205, USA; tprewitt@uams.edu; 4 Food and Health Lab, Department of Health & Human Development, Montana State University, Bozeman, MT 59718, USA; cbykershanks@montana.edu; 5 Department of Health Policy, Management, and Leadership, West Virginia University School of Public Health, Morgantown, WV 26505, USA; laandress@hsc.wvu.edu; 6 Department of Global Studies, University of North Carolina at Chapel Hill, Chapel Hill, NC 27514, USA; iosborne@live.unc.edu; 7 Department of Public Health, East Carolina University, 115 Heart Drive, Greenville, NC 27834, USA; jilcotts@ecu.edu

**Keywords:** rural, SNAP, food insecurity, food access

## Abstract

The Supplemental Nutrition Assistance Program (SNAP) is a critical program that helps reduce the risk of food insecurity, yet little is known about how SNAP addresses the needs of rural, food-insecure residents in the United States (U.S.). This study examines how rural, food-insecure residents perceive SNAP. Semi-structured interviews were conducted with 153 individuals living in six diverse rural regions of Arkansas, Montana, North Carolina, Oregon, Texas, and West Virginia. SNAP was described as a crucial stop-gap program, keeping families from experiencing persistent food insecurity, making food dollars stretch when the family budget is tight, and helping them purchase healthier foods. For many rural residents interviewed, SNAP was viewed in a largely positive light. In efforts to continue improving SNAP, particularly in light of its relevance during and post-coronavirus (COVID-19) pandemic, policymakers must be aware of rural families’ perceptions of SNAP. Specific improvements may include increased transparency regarding funding formulas, budgeting and nutrition education for recipients, effective training to improve customer service, connections among social service agencies within a community, and increased availability of automation to streamline application processes.

## 1. Introduction 

The United States Department of Agriculture (USDA) defines food insecurity as a “lack of consistent access to enough food for an active, healthy life” [1]. Food security is essential for health and development across the lifespan [2,3,4]. Food insecurity increases the prevalence and severity of diet-related chronic disease [3,5]. In the United States (U.S.), rural residents are more likely to experience food insecurity than urban residents. In 2018, the food insecurity rate in rural areas was 12.7% compared to 10.8% in urban areas [6].

Reasons for higher rates of food insecurity in rural areas can be attributed to food access. Distance to certain store types has been found to be associated with food insecurity [7] and other studies have found that very high food insecurity is linked with decreased consumption of fruits and vegetables [8,9]. In a South Carolina study, households with very low food security had lower odds of reporting access to affordable fruits and vegetables in their neighborhood compared to food secure households [9]. Additionally, very low food secure households had less variety and poorer quality of fruits and vegetables to choose from than food secure households. As in developing countries [10], climate-induced hazards such as flooding, hurricanes, and severe winter weather can make food security an even bigger challenge for rural, low-income populations [11].

To reduce food insecurity, the federal government created the Supplemental Nutrition Assistance Program (SNAP), formerly known as food stamps, in 1964 [12]. Overall, research shows that SNAP can help reduce food insecurity [13,14,15]. One study found that the SNAP reduced food insecurity by nearly 30% [15]. SNAP may improve health outcomes among participants, including both physical and mental health [16]. However, some studies reveal that SNAP recipients have poorer diet quality than non-recipients [17,18,19]. 

SNAP benefits can be used to purchase eligible food by lower-income individuals in the U.S. [20]. SNAP recipients can use their benefits on foods and beverages for consumption at home, but they cannot be used to purchase alcohol, tobacco, dietary supplements, or hot foods served in SNAP-authorized stores [20]. Generally, U.S. citizens with an income less than 130% of the federal poverty level and who pass a financial asset test are eligible for SNAP. The average monthly benefit—capped at $125 per person and $250 per household—is typically spent by most people by the end of the month [21]. In 2018, SNAP provided more than $68 billion dollars in benefits to approximately 40 million people [21].

Despite levels of poverty in rural areas and the need for greater food security, there are still barriers to enrolling in or purchasing food with SNAP benefits [22,23]. Many residents of rural communities report reluctance to use SNAP because of a heightened belief in self-reliance and a sense of shame if they do use such programs, and like urban residents, they have experiences of stigmatization when enrolled including negative vendor-client interactions [24,25]. Even with these barriers, 14.6% of rural households received SNAP benefits between 2008 and 2012, compared to 10.9% of metropolitan (urban) households and 13.8% micropolitan (small city) households [26]. 

Since SNAP is effective at reducing food insecurity, it is important to understand how rural SNAP recipients view the program. However, even with a growing body of research on SNAP utilization, there are gaps in the literature about how the specific population of food-insecure rural families perceive SNAP. To the authors’ knowledge, this is the first study to examine this topic in the U.S. Qualitative data, which provides a nuanced picture of factors that are often hidden in quantitative survey data, can illustrate a more comprehensive understanding of how rural families access and perceive federal nutrition assistance programs. Considering the dearth of research examining how rural residents perceive federal nutrition assistance programs, we demonstrate how racially and ethnically diverse, food-insecure rural residents across six states in the U.S. experience SNAP and its role in their lives.

## 2. Materials and Methods 

This study was conducted as a part of a joint project among members of the Nutrition and Obesity Policy Research and Evaluation Network (NOPREN) Rural Food Access Working Group [27]. The NOPREN Rural Food Access Working Group includes academic and non-academic researchers, public health and cooperative extension practitioners, and other experts in the field who focus on rural food access. To gain a better understanding of rural families’ perceptions of SNAP, researchers conducted semi-structured interviews lasting approximately 60 min with low-income primary caregivers in six rural counties across six states (Arkansas, Montana, North Carolina, Oregon, Texas, and West Virginia) in the U.S. 

### 2.1. Study Sites

Counties were selected based on geographic location, racial/ethnic breakdown, political representation (based on percent of House Democrats the state legislature), persistent poverty, and experience with recent natural disasters (see Table 1). To determine rurality, researchers used the Rural-Urban Continuum Code (RUCC)—and selected counties that had a RUCC of 4 or higher [28]. Two of the study sites have a majority African American population: Phillips County in Arkansas (63%) and Halifax County in North Carolina (54%), and two have high Hispanic/Latinx populations: Jefferson County in Oregon (20%) and Grimes County in Texas (23%). Political diversity ranged from Arkansas having 24% of their state legislature identifying as House Democrats to 58% in Oregon. At least three counties experienced persistent poverty and two states (North Carolina and Texas) faced recent natural disasters (Hurricanes Matthew and Florence for North Carolina and Hurricane Harvey for Texas). 

Researchers worked with partner organization(s) within each county to distribute flyers containing study information, and screen interested participants to ensure they were eligible for the study. Eligible participants (1) were the primary food shopper in the household, (2) served as the caregiver for at least one child under the age of 18 in their household, and (3) responded positively to at least one of the two-item food insecurity questions [29]. Researchers scheduled interviews with eligible participants at a time and place that was most convenient for them. 

Each interview was conducted by a trained study team member using a semi-structured, in-depth interview guide that was developed by the research team in partnership with Share Our Strength and Feeding America. This article focuses on interview questions related to the role of SNAP in mitigating household expenses and food insecurity, the experiences of rural participants with the program, what they would change about the program, whether they dropped out and why, and what would make it easier for people to participate in the program.

After the interview, participants completed a demographic questionnaire that included race, gender, education status, and program participation. The USDA’s 6-item food security screener was used when determining food security status [30]. All participants were compensated for their time with either a $25 gift card or $25 in cash (only Montana participants received cash). This study was approved by NC State’s Institutional Review Board (IRB), Study #16-654. Affiliated universities deferred to the NC State IRB as the IRB of record, with the following exceptions: Salish Kootenai College’s Institutional Review Board approved this study, IRB Protocol #2019_5_Dupuis_Shanks, and University of Arkansas for Medical Sciences, IRB Protocol #229190. 

### 2.2. Data Analysis

Interviews were digitally recorded and transcribed verbatim. To better understand rural families’ perceptions of SNAP, content analysis was used, to focus on barriers and facilitators to accessing the program. Content analysis is a qualitative approach that examines documents including written, verbal, or visual messages. The analysis strategy focused on the content of the language being used to describe SNAP, as well as barriers and facilitators to accessing the program. An advantage of content analysis is that it goes beyond code counting and focuses more on the interpretation and meaning of themes [31].

An iterative process was used to create the codebook. First, researchers created a codebook of deductive codes related to particular interview questions asked (e.g., barriers to enrolling in SNAP, program satisfaction, suggestions for improving the program). After interviews were transcribed, an inductive process was used to uncover themes within each code. After approximately one-third of interviews were conducted, all team members used the initial codebook to code four representative interviews (in teams of two) and added inductive codes that emerged from the data. Operational definitions were added to the deductive and inductive codes within the codebook. The PI (LHM) held a full-team codebook training where researchers discussed each of the codes and came to consensus on the final codebook. The final codebook was applied to all the interviews. Interviews were analyzed using the qualitative software program Atlas.ti version 7.0 (Scientific Software Development Gmbh, Berlin, Germany) to identify patterns and themes across interviews.

Code frequency (i.e., how often a code was mentioned by a participant in a transcript) for each interview was used to identify themes. Frequency of themes were calculated based on the number of times a theme was mentioned; this allowed researchers to count the same theme multiple times in one state and between states. Results were then organized by themes (grouped by codes within the consensus codebook).

## 3. Results

The study team conducted 153 interviews with rural food-insecure caregivers from six states. See Table 2 for demographic characteristics of study participants. Sixty-two participants reported they were Black or African American (40.8%), 25 (16.5%) Hispanic/Latino (16.5%), 39 (25.7%) White, 21 (13.8%) Native American, two participants preferred not to disclose their race (1.3%), and three participants identified with a race that was not listed (2.9%). The majority of participants were female (88.9%) with an average age of 37.9 years. The average household size was 4.16. More than 70% of participants (*n* = 112, 73.2%) received SNAP. Based on the USDA 6-item screener, 19.0% of participants reported having high or marginal food security, 49.0% low food security, and 32.0% very low food security.

Three main themes and eight sub-themes emerged from the interviews regarding the SNAP program. In the order of the frequency they were discussed, these themes were: (1) the adequacy of monthly SNAP benefit allotments; (2) benefits of participating in the SNAP program; and (3) barriers to effective program use. The following eight sub-themes were identified: (1a) stretching food dollars; (1b) confusion regarding monthly SNAP benefit allotments; (1c) fluctuations in SNAP benefits, (2a) provision of food to household communities; (2b) program features that rural families’ value; (2c) SNAP and diet quality; (3a) SNAP customer service; and (3b) SNAP application process. Table 3 describes each theme and its operational definition, as well as the total number of references for each theme.

### 3.1. The Adequacy of Monthly SNAP Benefits

The majority of participants who received SNAP benefits felt that the monthly allotment was not enough to feed their families. This sentiment was mentioned 75 times during interviews. As one female participant from North Carolina stated, “*I get food stamps, but I’m still not able to feed my whole household with those food stamps. I still have to put money in and then worry about how am I going to pay my rent ‘cause I’m putting money—you know what I’m saying? Like it’s just—it’s overwhelming*” (PID-NC18). As they addressed the shortages in SNAP benefits, families shared how they stretched food dollars, were confused about the monthly allotment, and dealt with the fluctuations in benefits due to changes in household employment. 

#### 3.1.1. Stretching Food Dollars

Families struggled to stretch their food dollars, reporting that the SNAP benefits they received were not enough to put food on the table for the entire month. However, multiple strategies were used to save money including buying food in bulk, shopping for discounted food, using coupons, growing/and or preserving their own food, accepting food from friends and family, and as a last resort, frequenting charitable food assistance programs such as food pantries. Even with food budgeting, some participants struggled to purchase food: “*It’s kind of hard budgeting with food stamps because at the end of the month and the beginning of the month, like right now my food supply is extremely low, so when it get extremely low it gets hard to budget*” (Female, Arkansas, PID-AR02). Food budgeting was mentioned multiple times as a way to address SNAP benefit shortages during the month. 

In order to stretch food dollars, families had to compromise on the kinds of foods they purchased, or sometimes get food from charitable food programs to make things work. After mentioning that they stopped buying as much “fresh stuff,” one female participant from West Virginia stated, “*Yeah, and so we just tried to make sure everybody was still fed but a different way, because we were excited about being able to pay our bills, so it’s kind of like we’re not months behind or asking for help from my mother-in-law, or my mother constantly for that. I mean occasionally I guess we would ask for food, but it’s easier to go to a food bank to receive some produce or boxed goods*” (PID-WV02).

Several participants were extremely careful in monitoring how much of their SNAP benefits they had used during the month, since having to pay out-of-pocket for food tended to put more financial stress on them. As one female participant in North Carolina commented, “*If I run out of stamps, I will just have to pay cash which is hurtful, because I did not realize how expensive food was without the SNAP card, but it’s pretty expensive. I done ran completely out and had to fill up my kitchen, buying groceries with cash and let me tell you, that’s something I don’t want to do, so I avoid it by any means necessary, but it will happen, because when you think about it, 30 days with a set amount of money for food, it could either last or it won’t*” (PID-NC09). One participant was informed by a SNAP administrator that she should not expect SNAP to cover her entire food budget for the month and to employ strategies to make her benefits last as long as possible.

#### 3.1.2. Confusion Regarding Monthly SNAP Benefit Allotments

Participants were also confused about how monthly SNAP allotments were calculated—several participants mentioned they did not understand why or how they received the amount of SNAP benefits were established. For example, when one interviewer in North Carolina asked what the participant would change to make SNAP better, she replied, “*Well I don’t know. What’s the purpose of why they give you what they give you? I don’t know the rules and the—I just try to, whatever I get, I try to make it last as much as possible*” (PID-NC26). Additionally, new employment and/or increases in wages resulted in decreases in SNAP benefits. As one female participant in North Carolina stated, “*I asked them (the Department of Social Services) because at one point I was getting like $600 and something. So why did it drop? Because now I’m only getting $400 and something. I still have the three kids. They’re telling me that my (pay)check goes up every year, but it didn’t go up but like $15. So why would that affect it?”* (PID-NC11). Participants expressed that variations in monthly SNAP benefits made it very difficult for participants to budget for upcoming living expenses.

#### 3.1.3. Fluctuations in SNAP Benefits

Families in this study with regular employment (29% were employed full-time and 22% were employed part-time), were often employed in very low-wage jobs. Participating in SNAP helped to extend their wages to pay for needs other than food for their families. Any decreases in SNAP benefits due to increased employment or wages prevented them from getting ahead because more money was then needed for food. A West Virginia female participant said, “*Minimum wage I think is like $8.75, so if you work that 40 h that’s really not enough to pay rent and bills*” (PID-WV04). Another working participant from Arkansas stated, “*I feel like families—parents that are working, they need help as well as the ones that aren’t working because they will disqualify you because you’ve made five dollars too much but they don’t know what you went through to make that five dollars. I see that they should—don’t look at just numbers, you know, because a lot of times those numbers mean nothing. They mean nothing because once you get through paying bills and providing the things that your children need and things for the household, a lot of times, you have nothing lef.”* (PID-AR26). Overall, participants that had SNAP benefits reduced due to increased employment or wages felt the reductions in monthly SNAP benefits were not proportional to their wage increases.

### 3.2. Benefits of Participating in the SNAP Program

For the rural families in this project, SNAP was a much-needed program to provide food for the household. SNAP was described as a key resource in providing food in the face of limited resources. As one female participant from Montana stated, “*I like that I don’t have to worry as much about my kids being fed. It would be impossible to feed them without this kind of thing*” (PID-MT17).

#### 3.2.1. Provision of Food to Household and Communities

Among the families in this study, there was a general recognition that other families in their communities needed SNAP, especially due to the lack of employment opportunities in rural areas, as described by one female Texas participant: “*If it wasn’t for the help from some of the EBT, the system, it would probably be hard trying to pay bills and everything with disability—by there not being as many jobs around here unless I was working. That is the only way that I could kinda provide and do better, but it’s kinda hard for me to work in my predicament. Because they don’t have a variety of jobs for handicapped people. I think everybody loves SNAP. Everybody who (is) on SNAP*” (PID-TX01). A male participant in Montana commented, “*10 years ago (I was) struggling during the recession. I didn’t have a steady job, I was in construction, and the construction economy tanked. And it was bad around here. … And I was working for the guys that had no work. So, we would scrape by with a job here or there or something like that, but—so, yeah, my children’s mother and I, when we were together, then she had the family on food stamps and my name was on there. It kinda benefitted us at that time*” (PID-MT06). Participants recognized that others around them were struggling, and in need of support from SNAP too.

#### 3.2.2. Program Features that Rural Families Value

Several features of SNAP were identified as contributing positively to the household in meeting food needs and reducing stress associated with lack of financial resources among the rural households in this study. For example, one participant in Montana talked about how receiving a set dollar amount for food each month helped him predict the monthly food budget and allowed his family to plan on those funds for food. This was also described by a female participant in Texas, “*I like the consistency (of SNAP). And not to mention it saved us from having to, like, even though his hours went down, we were still having to buy food. Once we got it, it saved the money that we were already putting into food. Plus, we were able to get more*” (PID-TX21). Participants appreciated that they could purchase a wide variety of food and beverage products with their SNAP benefits. Another participant in Montana commented, “*So many people don’t have access to food or don’t have the incomes to purchase food that it’s a good safety net that is needed*” (PID-MT08). The flexibility of the program to apply (or reapply) when household income fluctuated due to seasonal work availability was described as a benefit that could be counted on each year to buffer the reduction in wages. As one female in Oregon in said, *“We receive like four months of food stamps (each year) because that is when are eligible, but when my husband’s work increases, that is when we stop (Oregon)”* (PID-OR13).

#### 3.2.3. SNAP and Diet Quality

Several participants also shared how important the EBT card was to them in relation to their families’ diet quality. They noted that SNAP benefits allowed them to purchase fresh fruits and vegetables which they otherwise would not be able to afford. Participants expressed how they appreciated being able to shop at farmer’s markets using their SNAP benefits. Participants described being able to purchase more food, including healthier food, when they received more SNAP benefits, as illustrated by this exchange between an interviewer and a mother in West Virginia: “*When he [the husband] switched jobs we had enough money to pay at least all of our bills, there’s enough money now to put in the gas tank and stuff like that, but the food stamps dropped. So we were used to eating fresh stuff*” (PID-WV02).

Another North Carolina female participant described the importance of SNAP benefits when feeding her children: “If anything happens with me and my unemployment, and I go down there (DSS) and tell them that I’m a single parent and I have two kids, they’ll re-look at my case, open it back up, and then I’ll start back getting them. I don’t like being on assistance, but I just have it in place because my kids eat a lot, and I don’t want to see my kids go hungry because I was too selfish to go down there and ask for help” (PID-NC06). Other participants echoed that SNAP helped them purchase healthier food, which they felt was more expensive. For example, one male Montana participant said, “*The good thing about SNAP, other than commodities or a food bank, is you get to choose your food. Eating healthy is expensive … all that food stuff is cheap compared to eating right. Eating right costs money so, you know, the quality food comes with a price*” (PID-MT13). The cost of perishable foods and beverages was also brought up by a female participant in West Virginia who discussed struggling to give her son milk (his preferred beverage of choice). 

### 3.3. Barriers to Effective Program Use

While participants described SNAP as a key budget source and a critical lifeline, they also discussed two primary barriers to participation: customer service and the application process. 

#### 3.3.1. Customer Service

Several participants described being treated poorly by staff when applying or renewing their SNAP benefits. Some participants reported that SNAP program staff were unfriendly and participants felt they were being judged by staff. As one Montana participant commented, “*Honestly, it’s really hard to get a hold of anyone [at DSS] if you have questions about it [SNAP] or if you’re trying to renew it it’s really hard. I’ve waited on the phone one time—I think I was on hold for like three hours. Finally, somebody picked up and then I got hung up on and I was just like, ‘Forget it. It’s not even worth it.’ It’s frustrating*” (PID-MT17). Another participant from Montana stated, “*The only problems I’ve had with SNAP is just my interview parts with some of the people…They have signs up that say they’re there for you, that they’re there to work for you. There has been a lot of experiences where I’ve been there and the ladies have made me feel like I am just trying to get something from them, like I didn’t need it—I didn’t deserve it. You know, pretty much judging me. And, I mean, it just made me feel really bad, you know?*” (PID-MT13). In Oregon, one woman expressed concern that some in the community would not apply for benefits because they were afraid the staff working in the local office would deny the application based on personal dislike of the applicant. “*Some people say that they can’t get it (SNAP) because So-and-So works there and they don’t like them…that they don’t provide the service or they’ll decline it just for that reason*” (PID-OR10). Interactions with employees across the SNAP system seem to be fraught with stigma for SNAP recipients. 

#### 3.3.2. Application Process

Participants also talked about how the SNAP application process was time consuming, complex, and confusing. As one female participant living in Arkansas said, “*To be honest, I am looking forward to the day where there will be no longer a need for me to have any dealings with that particular program…It’s not anything bad. I don’t like applications. Here, within the past couple years, there’s been a lot of changes within that whole process and not necessarily a fan of the whole screening centers. I don’t understand that whole part, but that’s neither here nor there, I guess*” (PID-AR21). Another Texas female participant who was unable to re-certify for SNAP because of employment verification, explained her frustrations with the process: “*I tried to reapply, and they (DSS) wanted my employer to sign a form and fill it out. But my employer was based in Florida. I was kind of like a contractor. So, I didn’t have an employer here. I made phone calls. We did video interviews. But never face to face, so there wasn’t much I could do about getting them to fill that out. And I got declined because of it*” (PID-TX21). In Oregon, several participants voiced concerns regarding mixed documentation status households, even though the individual receiving SNAP was documented. Some participants did not understand the application process and there was a general misunderstanding of states’ policies regarding how to enroll in SNAP and how to qualify for the program.

## 4. Discussion

This study, conducted among 153 rural residents in six diverse counties, presents a context specific picture of food insecurity, and documents the importance of the SNAP for many rural families. It demonstrates that rural America is not a monolith [32], and policymakers and practitioners should be attentive to the ways families use and perceive SNAP across markers of race and ethnicity. Findings indicate that the SNAP is a crucial stop-gap and safety-net, that keeps a diverse set of families from experiencing persistent food insecurity, makes food dollars stretch when the family budget is tight, and helps them purchase healthier foods. For many rural residents interviewed, SNAP was viewed in a largely positive light. In fact, these families noted the need for additional SNAP benefits for themselves and the families in their surrounding communities. Overall, findings from this rural population are not unique compared to findings from urban communities. Including social stigma associated with participating in safety-net programs, negative interactions with staff administering or qualifying individuals for SNAP, and the lack of adequacy in SNAP benefits [24,25,33].

Discussions about rural America regularly center on the experiences of white residents, even though minority populations also live in rural areas [34]. The demographics of this study, comprised of 40.8% Black/African Americans, 16.5% Hispanic/Latinos, 13.8% Native American, and 25.6% white participants, offers a better understanding of how diverse rural communities in the U.S. experience food insecurity. While families explained how SNAP was a critical support, they also shared barriers that impacted their participation. These barriers can help policymakers and local implementing organizations to think critically about the services they provide, how they are perceived, and how they could be improved. For example, one frustrating element participants mentioned regarding SNAP is when wages increase, the decrease in monthly benefits can stymy attempts to save and build up assets for the future. 

Many participants in this study described the inadequacy of the funding amount for monthly SNAP benefit allotments. The amount of the monthly SNAP benefits received was frequently described as not being enough to meet families’ food budgeting needs. Other studies have suggested that SNAP monthly allotments should be increased due to the higher cost of food [35] and healthy food in particular [33]. As part of the Americans Recovery and Reinvestment Act of 2009 during the economic recession, SNAP monthly benefits were increased temporarily. One study evaluating the effect of the benefit increase on diet quality found that a 14% increase in SNAP benefits levels did not change significantly change diet quality [36]. However, a federally funded pilot program, the Summer Electronic Benefit Transfer for Children, that distributed additional monthly food benefits during the summer to low-income families found that receiving an extra $60 per month per child reduced the most extremely low food insecurity among children by 30% [37].

In agreement with other studies, low quality customer service and logistical difficulties with the application process were additional barriers that could be addressed within SNAP [38]. Issues with SNAP office-client relations have been documented in other studies [24], which underscore the need for greater communication and collaboration between public health organizations and SNAP recipients. During the SNAP application process, Hispanic/Latino families mentioned that mixed documentation status households were sometimes afraid to use the SNAP. Such fears and overall perceptions of stigma for participants in federal nutrition assistance programs can lead to greater food insecurity as individuals are less likely to enroll in SNAP when needed. 

Like all studies, several limitations are noted. While the study is geographically, racially, and ethnically diverse, only 25–28 individuals per county were interviewed. Therefore, this limits the generalizability of our findings and results should be interpreted cautiously. A larger, nationally representative sample is needed to further examine these issues quantitatively. Additionally, as part of the study’s eligibility criteria, while participants were screened for food insecurity, 19% had high or marginal food security. This is somewhat surprising since during interviews, participants were extremely vocal about their financial struggles and the coping strategies used to feed their families. Further research should consider examining the validity of food insecurity scales. Lastly, while there was an overwhelming response that SNAP monthly benefits should be increased, the questions regarding improving the SNAP could be considered leading questions.

## 5. Conclusions

SNAP is a critical safety-net program that helps reduce the risk of food insecurity among rural families. This study identifies barriers to participating in the SNAP that rural families face. The findings present a strong case for the continued need for SNAP. In efforts to continue improving SNAP, particularly in light of its relevance during and post-coronavirus (COVID-19) pandemic, policymakers and local implementing agencies must be aware of rural families’ perceptions of the program and listen to concerns in order to design and test improvements. Specific improvements may include increased transparency regarding funding formulas, budgeting and nutrition education for recipients, effective training to improve customer service, connections among social service agencies within communities, and increased availability of automation to streamline application processes. These improvements could be tested to determine the effectiveness in reducing food insecurity in rural populations using randomized controlled study designs. For example, SNAP participants could be randomized into a group that receives budget training and a more transparent and stepped approach to changes in benefits or a “usual care” SNAP group, to study the resulting changes in Food Insecurity in both groups. Finally, more research should be conducted to understand the demographic diversity of rural populations and how this diversity might influence experiences with food insecurity and food assistance programs like SNAP.

## Figures and Tables

**Table 1:** Study County Characteristics (N = 6).

State	County	RUCC Code	Persistent Poverty	Child Food Insecurity Rate (2016)	African American (%)	Hispanic/Latino (%)	Native American (%)	House Democrats (%)
Arkansas	Phillips	6	Yes	31.0%	63.0%	1.7%	0.0%	24%
Montana	Lake	6	No	21.2%	0.8%	4.1%	24.4%	41%
North Carolina	Halifax	4	Yes	26.8%	54.1%	2.6%	3.4%	38%
Oregon	Jefferson	6	No	24.2%	1.2%	19.8%	16.2%	58%
West Virginia	Calhoun	8	Yes	23.4%	0.2%	1.1%	0.1%	36%
Texas	Grimes	6	No	27.5%	17.1%	23.0%	0.2%	37%

**Table 2:** Study participant demographics (N = 153).

Characteristics	Total Sample	Arkansas (N = 28)	Montana (N = 25)	North Carolina (N = 25)	Oregon (N = 25)	Texas (N = 25)	West Virginia (N = 25)
Mean age	37.9	39.8	33.2	32.6	39.1	44	38.4
Mean number of adults in household	1.8	1.8	2.0	1.4	2.2	1.4	2.1
Mean number of children in household	2.4	2.3	3.2	2.2	2.5	2.0	2.2
Mean number of years living in county	25.5 years	37.0 years	19.3 years	18.2 years	19.8 years	32.7 years	23.9 years
Number (Percentage) of single parent households	67 (44%)	18 (64%)	9 (36%)	17 (68%)	2 (8.3%)	14 (56%)	7 (28%)
*Race/Ethnicity*							
Black/African American	62 (40.8%)	25 (89.3%)	0 (0%)	19 (76%)	0 (0%)	18 (72%)	0 (0%)
Hispanic/Latino	25 (16.5%)	0 (0%)	1 (4%)	0 (0%)	19 (76%)	4 (16%)	1 (4%)
Native American	21 (13.8%)	0 (0%)	21 (84%)	0 (0%)	0 (0%)	0 (0%)	0 (0%)
White	39 (25.7%)	2 (7.1%)	3 (12%)	6 (24%)	3 (12%)	2 (8%)	23 (92%)
Other	3 (2.0%)	0 (0%)	0 (0%)	0 (0%)	2 (8%)	1 (4%)	0 (0%)
Prefer not to answer	2 (1.3%)	1 (3.6%)	0 (0%)	0 (0%)	1 (4%)	0 (0%)	1 (4%)
*Education level*							
<8th Grade	10 (6.5%)	1 (3.6%)	0 (0%)	1 (4%)	7 (28%)	0 (0%)	1 (4%)
Some high school	22 (14.4%)	3 (10.7%)	2 (8%)	0 (0%)	8 (32%)	6 (24%)	3 (12%)
High school or GED	60 (39.2%)	13 (46.4%)	3 (12%)	15 (60%)	4 (16%)	15 (60%)	10 (40%)
Some college	41 (26.8%)	8 (28.6%)	12 (48%)	7 (28%)	3 (12%)	4 (16%)	7 (28%)
College degree	18 (11.8%)	3 (10.7%)	6 (24%)	2 (8%)	3 (12%)	0 (0%)	4 (16%)
>College	2 (1.3%)	0 (0%)	2 (8%)	0 (0%)	0 (0%)	0 (0%)	0 (0%)
*Marital status*							
Married/living with partner	62 (40.5%)	7 (25%)	9 (36%)	5 (20%)	22 (88%)	4 (16%)	15 (60%)
Never been married	53 (34.6%)	12 (42.9%)	10 (40%)	17 (68%)	0 (0%)	11 (44%)	3 (12%)
Divorced	19 (12.4%)	4 (14.3%)	3 (12%)	1 (4%)	2 (8%)	4 (16%)	5 (20%)
Separated	10 (6.5%)	2 (7.1%)	0 (0%)	2 (8%)	0 (0%)	4 (16%)	2 (8%)
Widowed	5 (3.3%)	2 (7.1%)	0 (0%)	0 (0%)	1 (4%)	2 (8%)	0 (0%)
Prefer not to answer	4 (2.6%)	1 (3.6%)	3 (12)	0 (0%)	0 (0%)	0 (0%)	0 (0%)
100% Federal Poverty Level or Less	96 (63.0%)	17 (60.7%)	19 (76.0%)	18 (72%)	12 (48.0%)	17 (68.0%)	13 (52.0%)
*Program Participation*							
SNAP	112 (73.2%)	25 (89.3%)	19 (76%)	21 (84%)	16 (64%)	18 (72%)	13 (52%)
Special Supplemental Nutrition Program for Women, Infants, and Children	50 (43.7%)	9 (32.1%)	8 (32%)	9 (36%)	10 (40%)	8 (32%)	6 (24%)
Free or reduced-price lunch or breakfast	107 (70.0%)	21 (75%)	18 (72%)	21 (84%)	21 (84%)	9 (36%)	17 (68%)
Free groceries or meals ^1^	50 (43.7%)	7 (25%)	9 (36%)	12 (48%)	10 (40%)	2 (8%)	10 (40%)
Food Distribution Program on Indian Reservations	2 (1.3%)	n/a	2 (8%)	n/a	n/a	n/a	n/a
Medicaid	126 (58.3%)	23 (82.1%)	24 (96%)	22 (88%)	22 (88%)	18 (72%)	17 (68%)
Temporary Assistance for Needy Families	16 (7.4%)	n/a	6 (24%)	3 (12%)	3 (12%)	2 (8%)	1 (4%)
WorkFirst	8 (3.7%)	n/a	n/a	5 (20%)	3 (12%)	n/a	n/a
Unemployment benefits	4 (1.85%)	n/a	n/a	2 (8%)	1 (4%)	n/a	1 (4%)
Social Security/Disability Benefits	27 (12.5%)	8 (28.6%)	2 (8%)	6 (24%)	1 (4%)	6 (24%)	4 (16%)
Other ^2^	47 (29.4%)	10 (35.7%)	6 (24%)	7 (28%)	1 (4%)	2 (8%)	6 (24%)
None	1 (0.7%)	n/a	1 (4%)	1 (4%)	2 (8%)	3 (12%)	7 (28%)
*Food Security Status*							
High or marginal food security	29 (19.0%)	5 (17.9%)	7 (28.0%)	5 (20.0%)	4 (16.0%)	2 (8.0%)	4 (16.0%)
Low food security	75 (49.0%)	15 (53.6%)	13 (52.0%)	7 (28.0%)	14 (56.0%)	14 (56.0%)	12 (48.0%)
Very low food security	49 (32.0%)	8 (28.6%)	5 (20.0%)	13 (52.0%)	7 (28.0)	9 (36.0%)	7 (28.0%)

^1^ The full question in the eligibility screener was: “Free groceries or meals from a food pantry, food bank, church, or other place that helps with free food.” ^2^ Other included specific names of programs.

**Table 3:** Themes, sub-themes, operational definitions, illustrative quotes, and code frequency, N = 153.

Theme	Sub-Theme	Operational Definition	Illustrative Quote	Code Frequency
(1) Adequacy of Monthly SNAP Benefits		Participant describes the adequacy of the funding amount for monthly SNAP benefit allotments.	“*Certain people they just don’t give enough benefits to, you know? Mine actually could be a little more, but I’m grateful for what I get. But, to me, it could be a little basic for one, or two persons it could be more than what they do. But like I said, I’m grateful for what I got because I could be getting nothing. And then I really would be struggling*.” (Female, Arkansas, PID-AR03)	136
	(1a) Stretching food dollars	Participant describes various strategies employed during the month to help cover their monthly food costs, including using coupons, bulk shopping, and budgeting.	“*What amount I get, I just make it last, you know. I just try to make sure that it’s not like, ‘Oh, we got food money. Let’s just go blow it,’ you know*.” (Female, West Virginia, PID-WV07)	46
	(1b) Confusion regarding monthly SNAP benefit allotments	Participant discusses confusion around how the monthly SNAP benefit allotment is calculated.	“*I could see that maybe they need to revamp the program for the elderly, that they would get a little bit more in food stamps ‘cause I know elderly people that only get, you know, 15, 16 dollars a month but they really don’t have the money to buy food ‘cause they’re having to buy other things, medications and things*.” (Female, West Virginia, PID-WV07).”	52
	(1c) Fluctuations in SNAP benefits	Participant describes fluctuations in monthly SNAP benefits due to certain situations, including wage and/or income changes or changes to members in the household.	“It’s only me because me and my wife separated. And I can pretty much deal with myself, you know, a little. But things can get tight because I was receiving food stamps at one time and then they cut it all the way back to $15 a month. But I still—I get that, but that $15 don’t go far.” (Male, North Carolina, PID-NC05)	38
(2) Benefits of the SNAP Program		Participant describes the benefits of participating in the SNAP program.	“*It’s a great program. It helps a lot and I’m so thrilled to be able to be part of one of them while I’m struggling*.” (Female, West Virginia, PID-WV13)	105
	(2a) Provision of food to household communities	Participant discusses the need for SNAP among families in their rural communities.	“*There’s a lot of babies, a lot of families out there…and I rather for someone else to have it that really needs it, than me having it, because I don’t need it as much anymore*.” (Male, North Carolina, PID-NC05)	23
	(2b) Program features that rural families’ value	Participant discusses features of the SNAP program that rural families’ value, such as consistency in when benefits are distributed.	“*Like I said, the government’s already helping you out with the benefits. I mean, what more can you really ask? It helps towards low-income housing families. Their adamant. They’re direct. And like I said, you can receive your benefits on time. If they say they’re going to be here that day, that’s what they mean*.” (Female, North Carolina, PID-NC04)	63
	(2c) SNAP and diet quality	Participant describe how changes in SNAP benefits impact their family’s diet quality.	“*He likes to have milk every day and then once the food stamps are gone it’s hard. I mean milk is something that if he’s wanting it every day, it’s not something that just lasts*.” (Female, West Virginia, PID-WV02)	19
(3) Barriers to Effective Program Use		Participant describes barriers to accessing the SNAP program.	“*When my husband got sick, we had a heck of a time trying to get ‘em [SNAP] because they were still looking at his income from months prior. And he hadn’t worked. Of course, he had money in savings, where we had been saving, but that was for the bills comin’ in. But they really didn’t care about that. All they seen was dollar signs and we could afford food, but we had to make sure the kids had roof over their head and water, electric, and heat*.” (Female, West Virginia, PID-WV23)	47
	(3a) SNAP customer service	Participant describes poor customer service when dealing with employees that process and re-certify SNAP applications.	“*They will find any little reason to kind of disqualify you*.” (Female, Arkansas, PID-AR26)	14
	(3b) SNAP application process	Participant describes frustrations with the SNAP application process including the length of time it takes to complete the application, complex questions, submitting the application, and/or lengthy application approval times.	“*If you do all the paperwork and everything when they tell you, like the dateline that they give you, it’s easy. But, if you miss that date where—it’s like really hard. You have to be like calling them and calling.*” (Female, Texas, PID-TX19)	33
